# A Conceptual Framework (2D-ME) for Explaining Self–first and Self–third Person Views of Prototyping Dynamics in Serious Games Design: Experimental Case Study

**DOI:** 10.2196/41824

**Published:** 2023-04-24

**Authors:** Sofia Hadjileontiadou, Sofia B Dias, Leontios Hadjileontiadis

**Affiliations:** 1 Department of Primary Education Democritus University of Thrace Alexandroupolis Greece; 2 Interdisciplinary Centre for the Study of Human Performance, Faculdade de Motricidade Humana Universidade de Lisboa Lisbon Portugal; 3 Department of Biomedical Engineering Khalifa University of Science and Technology Abu Dhabi United Arab Emirates

**Keywords:** 2D-ME conceptual framework, Technological-Pedagogical-Content Knowledge, TPACK, activity theory, AT, self–first and self–third person, Games TPACK, GTPACK, internal Activity Interview Script, iAIS, serious games prototyping

## Abstract

**Background:**

Design dynamics that evolve during a designer’s prototyping process encapsulate important insights about the way the designer is using his or her knowledge, creativity, and reflective thinking. Nevertheless, the capturing of such dynamics is not always an easy task, as they are built through alternations between the self–first and self–third person views.

**Objective:**

This study aimed at introducing a conceptual framework, namely 2D-ME, to provide an explainable domain that could express the dynamics across the design timeline during a prototyping process of serious games.

**Methods:**

Within the 2D-ME framework, the Technological-Pedagogical-Content Knowledge (TPACK), its adaptation to the serious games (TPACK-Game), and the activity theory frameworks were combined to produce dynamic constructs that incorporate self–first and self–third person extension of the TPACK-Game to Games TPACK, rules, division of labor, and object. The dynamic interplay between such constructs was used as an adaptation engine within an optimization prototype process, so each sequential version of the latter could converge to the designer’s initial idea of the serious game. Moreover, higher-order thinking is scaffolded with the internal Activity Interview Script proposed in this paper.

**Results:**

An experimental case study of the application of the 2D-ME conceptual framework in the design of a light reflection game was showcased, revealing all the designer’s dynamics, both from internal (via a diary) and external (via the prototype version) views. The findings of this case study exemplified the convergence of the prototyping process to an optimized output, by minimizing the mean square error between the conceptual (initial and updated) idea of the prototype, following explainable and tangible constructs within the 2D-ME framework.

**Conclusions:**

The generic structure of the proposed 2D-ME framework allows its transferability to various levels of expertise in serious games mastering, and it is used both for the designer’s process exploration and training of the novice ones.

## Introduction

### Background

Serious games are games that focus on learning while entertaining. As their design entails elements from both the game and learning design, it is a process of a rather complicated thread of decisions. Conceptualizations of this process contribute to serious game design modeling frameworks [1]. With regard to serious game designing, the work of Hunicke et al [2] proposed the Mechanics, Dynamics, and Aesthetics (MDA) framework. In particular, their work is fundamental in the area and realizes 3 distinct components that can be viewed from the user’s and the designer’s perspective. The Mechanics component describe specific options of actions that the user can perform while interacting with a game, and they can be described through verbs (eg, move). On the other hand, the Dynamics component refers to the combined player options toward a system of interactions at a higher level than the Mechanics component, that is, they can be considered the system-behavior of a game. However, the Mechanics component, being more specifically defined at a lower level of abstraction, is more reliable than the Dynamics component. Finally, direct and indirect interactions of the player with the game result in the Aesthetics component that refers to the emotional responses of the player (eg, fun), as they are provoked by the combination of the game Mechanics and Dynamics components, the latter bridging the Mechanics component with the Aesthetics component. Thus, a flow is realized during the game design, that is, an initial selection of the Mechanics component, then the Dynamics component, and finally the Aesthetics component. Although the work of Hunicke et al [2] was criticized as situational [3], it can serve as a direct framework to outline the serious game design. A detailed list of core mechanics was proposed by Järvinen [4] and a list of comprehensive Dynamics was proposed by Pendleton and Okolica [5].

Research in the area of serious game designing resulted in further frameworks that followed the work of Järvinen [4]. Moreover, Ávila-Pesántez at al [6] provided a systematic literature review regarding the methodologies, frameworks, and models applied to game designs in the period from 2008 to 2016, resulting in 11 approaches, whereas Viudes-Carbonell et al [7] provide a state of the art of 6 models in the area. However, the Learning Mechanics–Game Mechanics Framework [8] is worth mentioning, as it was based on the idea that learning occurs while interacting with the game, that is, Learning Mechanics can be mapped to the game’s Dynamics component and relevant thinking skills according to the revised Bloom taxonomy [9]. Thus, the learning objectives are diffused in the serious game design [10] to entail the game’s educational character. Following this approach, Pendleton and Okolica [5] proposed the Game Design Matrix structure, which reorientates the building block of the MDA framework [2] by first selecting the Dynamics component, according to the expected level of mastery in the Bloom taxonomy, so that the emphasis is initially given to the learning objectives that drive the learning outcome of the serious game. However, the design procedure is an iterative process and Hunicke et al [2] underline the importance of iterative analyses and refinement of the game design results. In a more detailed approach, Viudes-Carbonell et al [7], following the MDA framework, consider the game design as iterative cycles of design, test, evaluation, and redesign and stress the idea that the iterations would eliminate the risk of failure while designing a serious game. In particular, they focus on the prototyping iterations and consider them as series of small steps toward the enhancement of the quality of the game outcome. Following this path, Fullerton et al [11] consider prototyping an important part of the game design. Prototyping may take place by various means, for example, by pencil and paper, computer, and other artifacts in many disciplines (eg, engineering), to break out a complex problem to subproblems and work on these separately. However, a game is rule based; thus, it is sensitive to changes and/or prediction of possible impacts owing to rule changes. In this perspective, prototyping may resolve unpredictability issues in game design [12]. Moreover, considering games as pieces of art when compared with average software [13], their designing is even unpredictable as the design space may dynamically evolve through the emergence of new ideas and creativity.

The aforementioned approaches provide the frameworks of designing and establish steps for the enactment of design ideas. However, they do not refer to the knowledge that is needed for the materialization of the game design. With regard to this direction, the frameworks described in subsequent sections have been considered.

### Design Frameworks and Serious Games

#### The Technological-Pedagogical-Content Knowledge Framework

**Figure 1:**
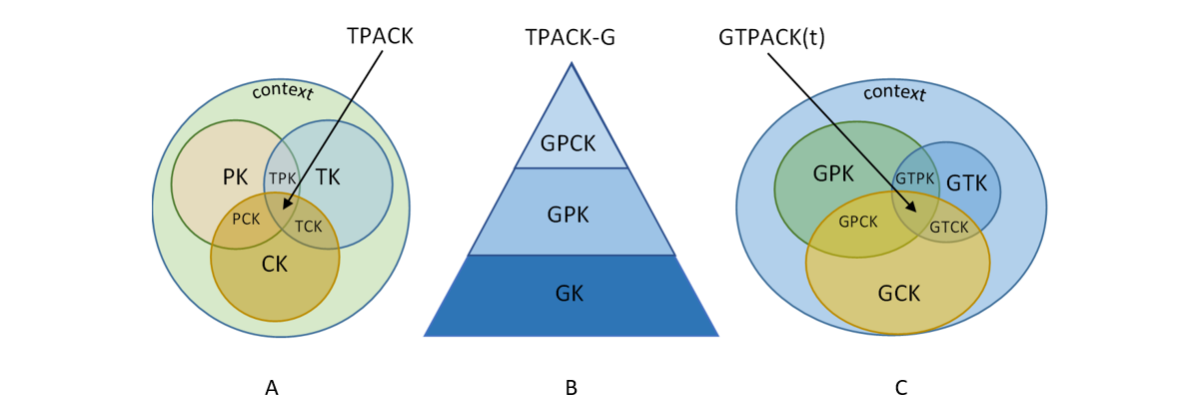
Schematic representation of the (A) Technological-Pedagogical-Content-Knowledge (TPACK), (B) Technological-Pedagogical-Content-Knowledge–Game (TPACK-G), and (C) Games Technological-Pedagogical-Content Knowledge (GTPACK; see the GTPACK subsection) frameworks. CK: content knowledge; GCK: game content knowledge; GK: game knowledge; GPCK: game pedagogical content knowledge; GPK: game pedagogical knowledge; GTCK; game technological content knowledge; GTK: game technological knowledge; GTPK: game technological pedagogical knowledge; PCK: pedagogical content knowledge; PK: pedagogical knowledge; t: dynamic; TCK: technological content knowledge; TK: technological knowledge; TPK: technological pedagogical knowledge.

The Technological-Pedagogical-Content Knowledge (TPACK) was proposed as a framework to account for the knowledge needed by the teachers to use information and communications technology in their classrooms [14-16]. In particular, this framework extended the notion of Shulman [17] concerning the pedagogical content knowledge (PCK), by foreseeing the inclusion of knowledge related to technology. Figure 1A depicts the TPACK framework and manifests the interplay between forms of knowledge (content knowledge [CK], pedagogical knowledge [PK], technological knowledge [TK], PCK, technological PK [TPK], and technological CK [TCK]). More specifically, CK refers to the knowledge of the content of the lesson, for example, the concepts that are to be taught; PK refers to the pedagogical considerations as to how to teach the content; TK refers to the knowledge about the technological affordances and their use; TCK and TPK refer to the way the technology could support and materialize the delivery of content and pedagogy; and finally, PCK refers to the pedagogical way in which the content could be delivered to the learners. The contextualized synthesis of all these forms of knowledge constitutes the core TPACK framework. TPACK is usually presented as 3 overlapping equal cycles denoting 3 forms of knowledge (CK, PK, and TK) and their overlaps (TCK, TPK, PCK, and TPACK), all included in a circle that denotes the knowledge that is needed for the integration of technology in a relevant sociocultural context. TPACK has been widely used as a framework to reflect information and communications technology integration in the classroom even under education emergency conditions [18].

#### The TPACK-Game Framework

TPACK serves as a theoretical reference framework of necessary knowledge for the technology integration in different contexts; for example, Foster et al [19] proposed TPACK as an aid for the teacher when selecting a game to realize its constraints and affordances for technology, pedagogy, and content. However, Willermark [20] commented on TPACK for its abstract reference to general technology that could be used in the classroom, that is, it lacks the specificity that might contribute to further specification of knowledge that is needed. In this regard, in the specific case of computer game integration in the classroom, Hsu et al [21] proposed TPACK-Game (TPACK-G; Figure 1B) to reflect the knowledge that the teachers need in this case. In particular, they define game knowledge (GK) as the knowledge about the general use of computer games, game PK (GPK) as the pedagogy related to the way a game is used in the classroom, and game PCK (GPCK) as the “knowledge of using games to implement teaching methods for any targeted content” [21]. On the basis of prior work on the developmental pathway of TK and TPK to TPACK, Hsu et al [21] proposed the GPCK as a pyramid, where GK constitutes the basis, followed by the GPK, and finally, at the top, the GPCK. Upon this modeling, they claim that for the development of specific GPCK, the GK and GPK are prerequisites, so a lack of the foundation knowledge of how to play a game, that is, familiarity with gaming environment (GK), leads to a lack of GPK and therefore GPCK [21]. Structural relationships were also detected between the TPACK-G attitudes toward games and actual teaching use [22].

Both the TPACK and TPACK-G frameworks are proposed through a “stationary-like” approach, whereas a more dynamic one might reveal the evolution of types of knowledge over time while an activity takes place, for example, during the iterative approach toward the designer’s activities while designing a serious game. The activity theory (AT) framework may provide a lens toward this direction, as explored in the subsequent section.

#### The AT Framework

##### Overview

**Figure 2:**
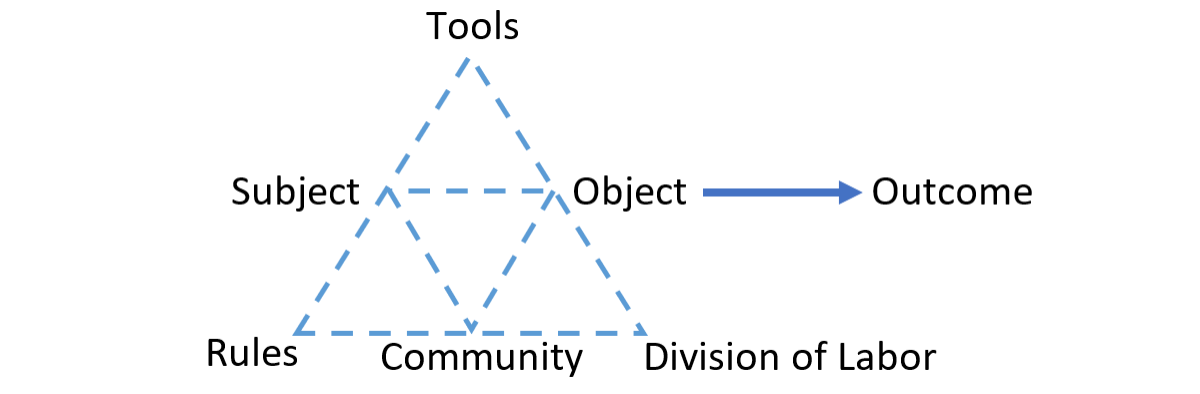
Schematic representation of the main constructs that form the activity theory framework.

The AT framework has its roots in the 1920s and provides a conceptual framework according to which, the human activity connects the individual world of the subject with the social one (Figure 2). Upon its initial formulation [23,24], the AT framework focused on the activities of the subject, for example, human being. For the subject to meet his/her needs, he/she performes activities by interacting with objects in the world by means of tools that mediate the interaction. Thus, the subject is involved in an activity motivated by an expected outcome (the “why” of the activity), pursues goals that direct the activity (the “what” of the activity), and performs operations (the “how” of the activity) [25]. However, the connection between subjects and activities is bidirectional as they mutually determine one another. Basic principles of AT have evolved through the works of Leontiev [23,24], namely [26] (1) object orientedness, which states that objects differentiate the activities that are directed to them upon motivation toward a need; (2) hierarchical structure of activity, which is a hierarchical system that shows the dynamic relationship among 3 levels of human activity. From a top-down perspective of the activity, at the top, the activity driven by the motive is considered. However, the activity in a lower level is performed through conscious actions that are directed to goals (possibly decomposed to subgoals) that are to be undertaken to fulfill the object. At an even lower level, actions are performed through unconscious operations that are orientated to conditions under which the goal is to be reached; (3) tool mediation, toward a purposeful activity; (4) development, as a context to analyze the activity, for example, from the research respective, this leads to the analysis of the dynamics of the object transformations over time; and (5) internalization externalization.

##### Synergies of AT in Serious Games Prototyping

In the work of Carvalho et al [27], the AT framework was used for the analysis and design of serious games. In particular, they proposed the AT-based conceptual model of serious games, which contributes to the realization of the components of a game and their roles and to the recognition of the educational objectives. In addition, they foresee 3 activities: the gaming, the learning, and the instructional activity and their tensions to describe the contexts in which the game is used. Moreover, for each activity, they propose elements taxonomized into actions, tools, and goals per activity, upon which a game may be analyzed or designed. However, the AT-based model of serious games is restricted only at interactions between the triad, that is, subject, object, and tools. The works of Viudes-Carbonell et al [7] and Manker et al [28] use the AT approach to propose analyses of the prototyping procedure during the design of a serious game. In particular, Viudes-Carbonell et al [7] focus on the iterative character of the serious game design and present an early-stage methodology and a prototype for learning a case-concept. Moreover, Manker at al [28] also focus on the game prototypes and among other, on the role of the prototype to the internalization and externalization of the designer’s ideas. The combination of the AT framework with the TPACK framework, namely TPACKtivity, has been proposed by Terpstra [29] as a lens to follow preservice teachers’ (PTs’) TPACK development through their activities, that is, realizing its dynamic character across the time.

##### Contradictions, Social and Relational Self-views Within AT

The traditional logic considers contradictions in a system as problems; dialectical logic, however, considers that learning and development are driven by them. Hence, within the AT framework (Figure 2), analysis of the trajectory of the object across time can contribute to the realization of the evolution of its current existence and the expected contradictions that may drive its further development [26]. Thus, a deep assumption is that the activity develops across the time mainly through contradictions that arise, that is, upon resolving 1 contradiction, another may arise. Continuous approach of the activity provides continuity in viewing the driving forces (triggers and resolutions of contradictions) that steer this activity further. Focusing on the subject of the activity system, initial ideas about the dichotomy of the context-free information processing of human functioning as opposed to society were further elaborated, extending the subject approach in the AT. In this direction, Stetsenko and Arievitch [30] proposed a framework to combine the social and relational view of the self. In their approach, the subjectivity that is developed outside and in the activity system of reference, may result from the role according to the social position of the human being (ie, serious game designer), which along with social interactions and collective practices coevolve. On the other hand, the self is considered as an active agentive role that an individual does not only acquire historical cultural norms and experiences but also develops them further through change and novelty. In this sense, the ever-expanding social practices entail human subjectivity, and both social and relational views of the self, coexist, or self-coexist among planes of the activity through the internalization and externalization processes.

The aforementioned design frameworks present either the design of a serious game through the lens of the AT framework or the combination of TPACK with the AT framework. However, they hardly focus on the subject of the activity (ie, the designer), and when they do so, a macrolevel is adopted, referring mostly to the procedures per se, for example, the connection of the subject’s internal plane with the world’s plane.

### Study Aims

In this paper, we introduce the 2D-ME framework, an innovative conceptual framework that adopts a microlevel approach toward the serious game designer’s inner world while he/she is prototyping. In particular, we dynamically followed the designer’s first- and third-person views (2D-ME) during the prototyping activity under the lens of the AT framework and an extended version of TPACK in the game design area, namely Games TPACK (GTPACK). Under this aim, the following research questions were investigated:

1. Is 2D-ME a useful framework for describing the prototyping dynamics during the design of a serious game?
2. How do the internal (first person) and external (third person) views facilitate the optimization of the prototyping output during a serious game design?

Although we refer to the serious game designer, the 2D-ME framework could easily be transferred to the case of serious game developer (sometimes these 2 roles are undertaken by the same person). Moreover, as it offers new insights in the internal processes during prototyping, the 2D-ME framework could also be used as a research framework for analyzing the research aspects of dynamics during creative prototyping.

Despite the conceptual character of the proposed 2D-ME framework, its practical implementation can easily be realized via a series of steps:

1. Conceptualization of the initial idea target of the serious game
2. Identification of the AT constructs
3. Activation of a metacognitive process (eg, reporting in a diary via a self-interview script), which could facilitate the interplay between the roles of first- and third-person view during the prototyping
4. Enabling and capturing of the dynamics between the AT constructs
5. Adaptation of the AT dynamics for the convergence of prototype successive versions to the initial idea target of the serious game

## Methods

### The Proposed 2D-ME Conceptual Framework

#### Main Constructs and Dynamics

**Figure 3:**
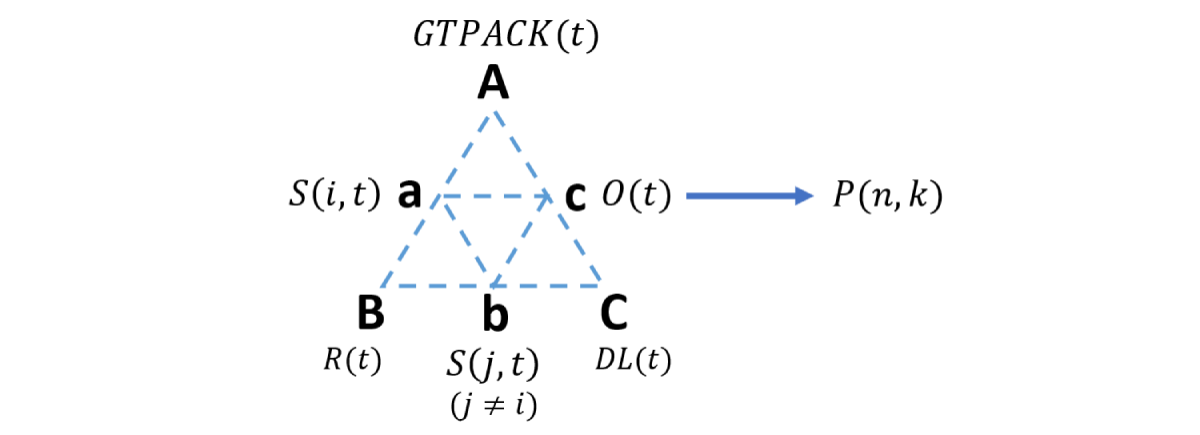
Schematic representation of the main constructs that form the 2D-ME framework. DL: division of labor; GTPACK(t): Games Technological-Pedagogical-Content Knowledge (dynamic); O: object; P: prototype; R: rules; S: subject.

Stemming from the design frameworks presented so far, the proposed 2D-ME conceptual framework is described in this section. Figure 3 depicts the main constructs of the 2D-ME framework. As it is apparent from the comparison between the shapes of Figure 2 (AT) and Figure 3 (2D-ME), a connection of the 2D-ME framework with the TPACK, TPACK-G, and ΑΤ is noticeable. This is materialized by the inclusion within the 2D-ME framework of (1) the concept of triangles and interactions as in AT and (2) the combination of TPACK with the TPACK-G as a new entity in the AT triangle, namely GTPACK. However, all constructs of AT (Figure 2) are replaced with new ones (Figure 3), as follows:

- Instruments or mediating artifacts → *GTPACK(t)*; *t=1,2,...*
- Subject → Subject *S(i,t), i* =first-person or third-person view; *t=1,2,...*
- Rules → Rules *R(t); *t*= 1,2,...*
- Community → Subject *S(j,t), j*=first-person or third-person view; *j≠i*
- Division of Labor → Division of Labor *DL(t)*
- Object → Object *O(t), t*= 1,2,...
- Outcome → prototype *versions P(n,k), n=1,2,...,N; k=1,2,..., M* (last version)

As it is apparent, the 2D-ME framework embeds within its structure a dynamic activity within its constructs that interact and (potentially) are modified across time (*t*). In particular, the prototype designer is considered as the subject *S*(*i,t*) and subject *S(j,t), j≠ i,* alternating across time (*t*) between the first-person and the third-person views. The *S*(*i,t*), motivated toward the materialization and optimization of the object *O*(*t*), that is, the game prototype *P(n,k), n = 1,2,...,N; k = 1,2,...,M,* at specific time instances *k*, uses their current *GTPAK* (*t*) to produce each prototype version *(P(:,1), P(:,2),...)* toward *P*(:,*M*), which is the final version of the outcome of the activity system. It should be noted that the *n* parameter expresses the different dimensions of prototype (see *The Object Outcome [Prototype]* section). Apparently, the first-person and the third-person view of the subject communicate upon rules *R*(t) and division of labor *DL*(t).

As it can be deduced from the aforementioned 2D-ME structure (Figure 3), the flexibility of the AT framework to project the activity at different levels of analysis is used here to shift from a macro- to a microscale approach. More specifically, at the macroscale approach, the prototyping procedure in the game design provides samples of the evolution of the game design across the time. From a main concept perspective, the 2D-ME framework adopts a microscale approach by following the inner iterative prototyping procedure that follows the game designer across the time. Upon this, an AT triangle is defined, in which the subject, that is, the designer of the serious game, is the focus of the approach. The 2D-ME framework foresees the designer to adopt 2 perspectives while prototyping, that is, the first-person and third-person views. In this way, 2D-ME follows the internalization and externalization actions within the game designer and provides a conceptualization of the prototype design before its externalization to the others (collaborators, users, etc). To further explain the main concept, a description of each entity of 2D-ME is presented in subsequent sections.

#### The GTPACK

The GTPACK (Figure 1C) is considered in 2D-ME as a tool that extends the TPACK in the area of the necessary knowledge base for the serious game designing. In particular, the Game CK refers to the cognitive content of the game, that is, the subject matter of the discipline of reference. Game Technological Knowledge refers to the knowledge base relevant to the technology related to the games design and its use. Finally, Game PK reflects the knowledge of possible misconceptions, learning theories and how to apply them in games, metacognition, etc. On the basis of this perspective, in a game designing procedure, Game Technological CK might be triggered for the representation of the concept to be learned in the game environment, the GPCK might be triggered for the definition of the game dynamics, and the GTPK might be triggered for the selection of the game mechanics, whereas the GTPACK might be triggered for the whole game design and aesthetics. The GTPACK serves as a tool that mediates the activity of the subject upon the object toward the outcome. It should be noted that the GTPACK is not a stable framework across design time, as the designer might interact with resources (either human and/or material) that may alter its load and dynamics across the time.

### The Subject

The 2D-ME framework proposes 2 points of view of the subject, the first-person and the third-person view, considering transitions from the internalization to externalization processes and vice versa, yet both defined within the same person. In particular, the first-person view considers the subjective version of the subject in the activity system that reflects his/her activity following rules of designing a serious game, that is, the social world. The third-person view considers the self-version of the subject that reflects his/her activity according to the object of the activity system that he/she acts upon as the designer. The third-person view is the agentive self who leads the activity as he/she are engaged with the social world. On the basis of this perspective, the first- and third-person views hold a GTPACK and they both coevolve with the activity, yet the third-person view is mainly reflective, to actively introduce possibilities; enhance them by prioritizing other actions; and possibly generate activities that stem from the reality, fantasy, and serendipity.

### The Rules

In the AT framework, the rules primarily mediate how the subject acts in relation to the object, including the tools used and the ways they are used [31,32]. This could include specific and/or well-established patterns of behaviors that could be followed because of either cultural norms and/or other reasons (eg, professional and legal mandates). In 2D-ME, the rules refer to the ways of communication between the first- and the third-person views during the subject’s communication. Apparently, this interpersonal communication follows some norms (rules) that facilitate the process by which the subject is engaged in unspoken internal dialogue among different and sometimes conflicting attitudes, thoughts, and feelings, all represented by the first- and third-person views. Clearly, these rules are subject dependent and can be imposed, sustained, redefined, modified, or dropped within a dynamic process that could add to the metacognitive skills of the subject.

### The Division of Labor

The main responsibilities (what is being done by whom) that are involved toward the object define the division of labor in AT, also taking into consideration the horizontal division, that is, across tasks, and the vertical division, that is, across the power, positions, access to resources, and rewards [31,32]. In 2D-ME, the division of labor refers to the contribution of 2 subject’s views, that is, the first- and third-person views, toward the object within the interpersonal communication. The concept of horizontal and vertical division can also be transferred at this inner level by considering the different aspects that could be triggered during the interpersonal communication. For example, the horizontal division could include a systematic definition of a sequence of interpersonal sessions across the prototyping process. Moreover, the vertical division could prioritize the first-person view in the access to and use of technological resources (eg, game developing software), leaving space for the third-person view for reviewing the aesthetics (eg, rewarding and/or corrective feedback on game sensation, fantasy, or challenges).

### The Object Outcome (Prototype)

The object in AT can be approached by various views, that is, the object is seen as a thing to be acted upon, an objectified motive, or a desired outcome [33]. The object in 2D-ME is to materialize an idea toward optimizing the outcome, which is a prototype of the game. The prototypes could vary in purpose, as they can refer to a role, an implementation approach, a look, or a sense of feeling. These include interaction with the users (role), the construction of the game (implementation), and users’ experience (look and feel) [21]. This multiplicity in roles results in multiple dimensions (denoted with the parameter *n*, with *N* being the total number of dimensions). The various versions of the prototype in 2D-ME (ie, *P(n,k), n = 1,2,...,N; k = 1,2,...,M*) are tangible externalizations of the development transformations that the activities undergo between the constructs across the serious game design process.

### Prototyping Optimization

**Figure 4:**
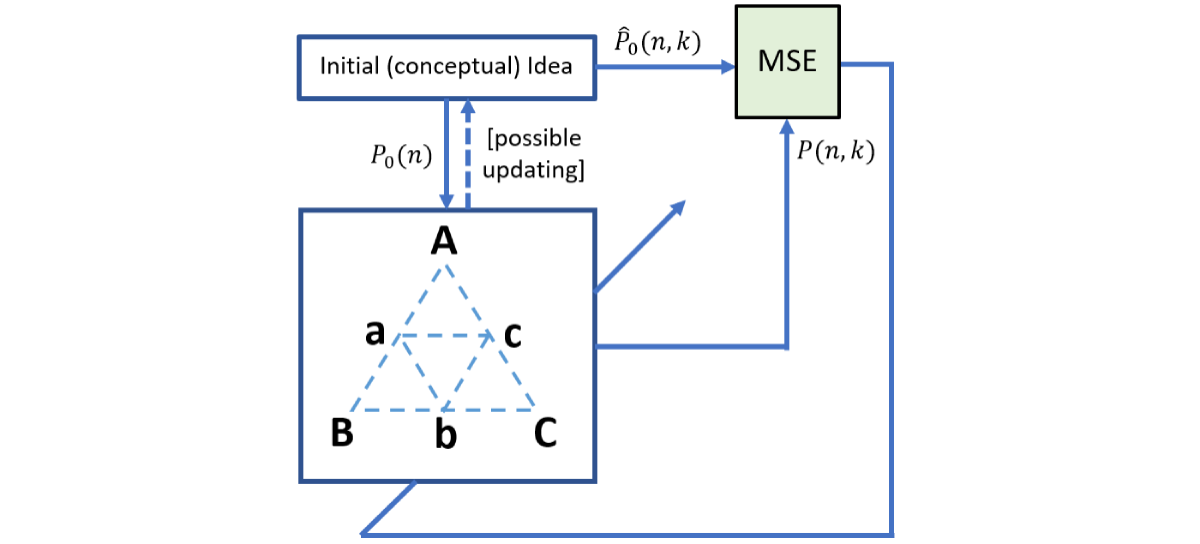
Schematic representation of the prototyping optimization process in 2D-ME framework. The constructs triangle (Figure 3) is used as the adaptation engine toward the minimization of the difference of the conceptual (initial and updated) idea of the prototype <inline-graphic xlink:href="games_v11i1e41824_fig10.png" mimetype="image" xlink:type="simple"/> and its current version (*P(n,k)*), that is, gradually reaching to the optimization of the prototype (at *k=M*) across its intermediate versions (at *1≤k≤M*) and across its dimensions. MSE: mean square error.

The proposed 2D-ME conceptual framework considers the prototyping process as an optimization process that converges to the optimal prototype (*P*(*N*,*M*)) by minimizing a cost function. This is clearly depicted in Figure 4, where the constructs triangle (Figure 3) is used as an adaptation engine. The cost function per *k* is the convex function of the mean square error (MSE) between the conceptual (initial and updated) idea of the prototype 
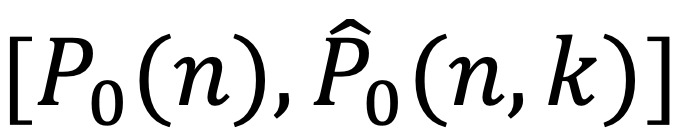
 and its current version (*P*(*n*,*k*)) is presented as follows:



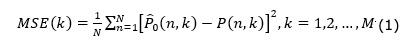



The adaptation engine provides the convergence, so the whole activity gradually reaches to the optimized outcome, that is, *P*(*N*,*M*). However, the optimization convergence is not seen within an absolute perspective but rather from the designer’s view and experience. This means that the current optimization process could stop at a point that the *P*(*N*,*M*) will not express the full spectrum of the designer’s skills and knowledge, but the process will reach at a point where the designer has already concluded for the validity of the final output and the sufficient use of their knowledge and experience, that is, their current level of GTPACK.

Furthermore, as it can be seen from Figure 4, the initial idea *(P_0_(n); n = 1,2,...,N)* acts as a trigger to the adaptation engine and, at the beginning of the process, it is considered as static. However, the processes within the adaptation engine (eg, the interpersonal communication), apart from the update of the version of *P*(*n*,*k*), could potentially affect the initial idea, as well. In this vein, its transformation, that is, 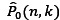, is considered at the next stages of the design process. This transformation could alter specific dimensions of the prototype (eg, eliminate some and add new ones) and/or the characteristics of each dimension. Apparently, the frequency of the changes of the 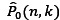 is anticipated to be low, considering the level of the designer, that is, how clear and solid is the initial idea in his or her mind. To this end, during the optimization process, some versions of the 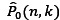 could be constant across *k*, for example, 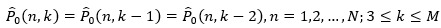. This is, usually, anticipated as the prototype converses to its optimum version, because the designer has already a clear view of its intended and the constructed version.

### Capturing of Dynamics

From what it is presented so far, it is clear that 2D-ME entails dynamics among its constructs and in the prototyping optimization process. To capture such dynamics, some sampling tools should be used. Clearly, the interpersonal communication (between the self–first person and the self–third person) that is involved in 2D-ME sets the highest hurdle in the dynamics capturing process. One way to infer for the activation of such communication could be the use of a systematic approach that would include a subject’s diary. The latter could be structured on specific contextual factors reflected in a set of statements. A characteristic example of such statements is included in the activity checklist [34,35], which is based on the AT. As the activity checklist provides a “contextual design space” [28], this could be adapted, accordingly, to include statements that could help the designer to describe in his/her diary the dynamics used between the 2D-ME constructs. In this way, the activity checklist itself can serve as a “valuable aide memoir and a tool for reflexivity” [36]. Nevertheless, the activity checklist contains many items (43 for design and 37 for evaluation) [35] that make its full use quite difficult in practice. As a remedy, adaptation of the activity checklist to the form of interview questions, limited in number, was proposed by Duignan et al [37]. Hence, this interview form of activity checklist could trigger reflection and self-talk and could assist in capturing specific dynamics at the various phases of the prototyping process. In this vein, internal questions related with the 2D-ME dynamics that could be self-answered and documented in the diary could be formed, as an internal Activity Interview Script (iAIS), tabulated in Textbox 1.

An additional source of understanding the dynamics is via the exploration of the alterations across the different versions of the prototype. Apparently, this is a lower quality sampling of the dynamics when compared with the aforementioned approach; it provides, however, a way of monitoring the dynamics, especially when exploring the differences across the dimensions (*n*) of the prototype versions across the design time. A process of deconvolution is then applied to identify the constructs that were more frequently used in a current version of the prototype. Projecting this construct activation across the whole prototyping process, the dynamics per construct can be revealed.

**The internal Activity Interview Script.**

**Question 1. Goals-related questions**

- Question 1.1. What are the different roles that you identify in yourself and are involved in the prototyping?
- Question 1.2. How do you breakdown, in a step-by-step form, your prototyping process?
- Question 1.3. How this fine-graining process can vary?
- Question 1.4. How do you know that you have successfully completed each intermediate goal?
- Question 1.5. How could you evaluate the achievement of your higher-level goals?

**Question 2. Contradictions-related questions**

- Question 2.1. What contradictions can you identify between your different roles?
- Question 2.2. How do you resolve such contradictions?

**Question 3. Tools, transition, internalization, externalization–related questions**

- Question 3.1. How does Games Technological-Pedagogical-Content Knowledge support the transition between the first-person and third-person view?
- Question 3.2. How does Games Technological-Pedagogical-Content Knowledge affect the way you think and reason about the prototyping activity?
- Question 3.3. How difficult is to perform the different interpersonal roles?
- Question 3.4. How do you use representations of your work between the different interpersonal roles?
- Question 3.5. How do you internally handle the complexity of the prototyping process?

**Question 4. Rules, division of labor–related questions**

- Question 4.1. How do explicit or implicit rules, norms, and procedures affect your different interpersonal roles?
- Question 4.2. How do you organize the different interpersonal roles across the prototyping process?

### Experimental Case Study Setup

An experimental case study was set up as a running example of the realization of 2D-ME in practice. In particular, the case refers to a PT as the designer of a serious game for kindergarten through grade 12 students. The motivation for the latter was drawn from the physics student’s exercise textbook (page 63) for the fifth grade for primary school in Greece. In the latter, an exercise related to the light reflection on mirrors presents a situation in which a series of mirrors are fixed at different positions in a box. At the perimeter of the box there are 4 openings where specific objects (compass, scissor, and pencil) and a boy’s eye are positioned. The student is asked to identify and draw the paths of the light reflections on mirrors so as the boy can see the 3 objects (see *P_o_*(*n*) in Figure 6). This exercise was presented to the PT as a stimulus for the design of a related serious game on light reflection. The PT was at his final semester of his studies at the Department of Primary Education, Democritus University of Thrace, Greece, and he already had attended 1 semester concerning serious game design using Scratch [38]. In particular, concerning his GTPACK, the PT had Game CK about the light mirror refection, which was the concept to be practiced by the serious game. Moreover, he had extended GPK because of his studies and good Game Technological Knowledge based on the familiarity with the game prototyping, design, and implementation in Scratch. Thus, his current GTPACK was considered adequate for this experimental study. It should be noted that the PT was already aware about the GTPACK framework.

**Figure 5:**
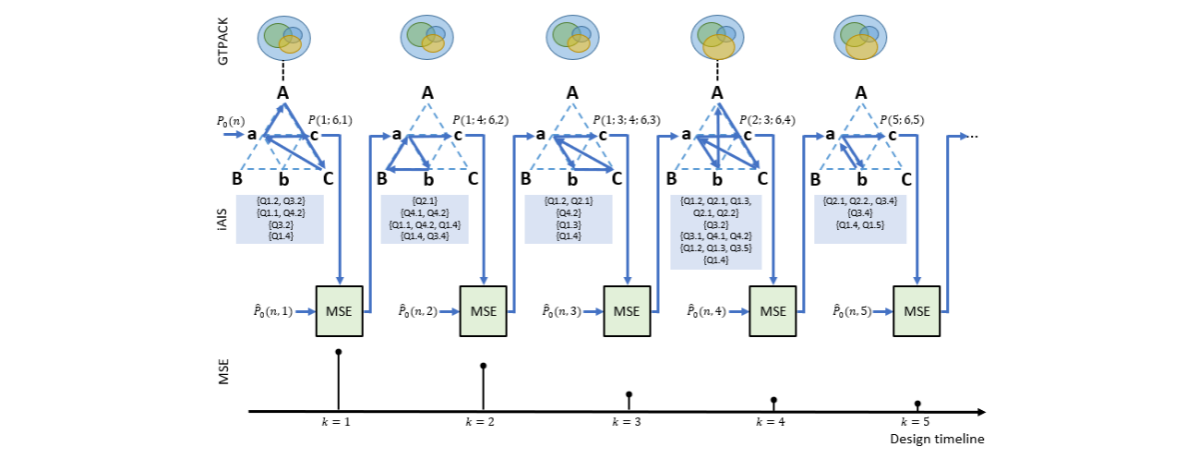
Schematic representation of the dynamics across the prototyping optimization for *k=1,2,...,5*. At the top, the Games Technological-Pedagogical-Content Knowledge (GTPACK) alteration across are displayed, where the dynamics affect its internal set (at *k=1,4*). The constructs dynamics are denoted with arrows, accompanied with the related internal Activity Interview Script (iAIS) questions (Textbox 1). The optimization process is depicted for each outputted prototype, whereas the estimated mean square error (MSE) is depicted at the bottom, showcasing its reduction as the game design evolves.

The research design foresaw controlled experimental situation that took place at the Democritus University of Thrace computing laboratory within 1 month. The PT willingly participated in this experiment and was informed about the experimental process before embarking on it. More specifically, he was asked to express in a diary the conceptualization of his initial idea *P*_0_(*n*) of the serious game and then to proceed to his prototyping activity and optimization (Figures 4 and 5). Moreover, he was addressed with the aforementioned iAIS and was advised to document his relevant thoughts in the diary during the prototyping procedure. It was made clear to the PT that the use of the diary should be spontaneous and in no way should hamper his creative impetus. Furthermore, he was informed and consented that an observation documentation would be made by a researcher (first author, SH) to capture the explicit prototyping performance of the PT. At the end of the experiment, the PT consented to openly share the diary; a thematic analysis of the diary was manually conducted and combined with the data from the observations along with the outcome of the whole activity.

**Figure 6:**
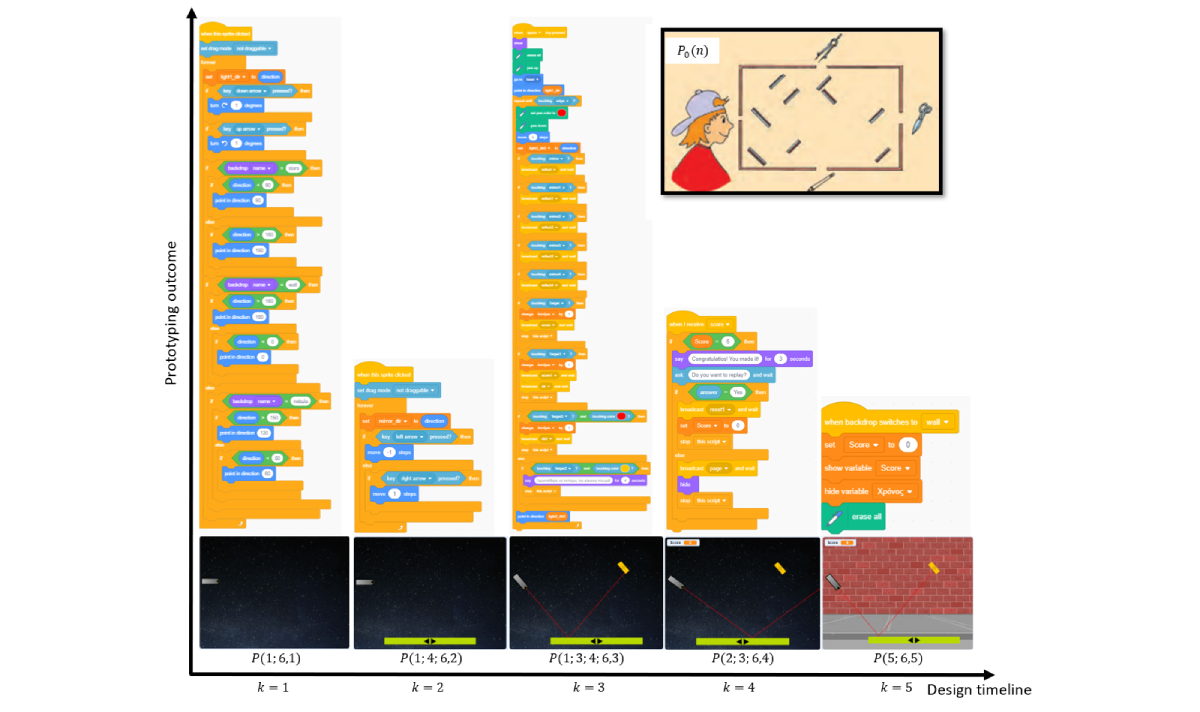
Illustration of the produced game prototype scenes, along with the addition of the Scratch programming code at each *k*, *k=1,2,...,5*. The initial idea, *P*_0_(*n*), that acts as the stimulus for the creation of the light reflection game is also depicted.

## Results

### Conceptualization of the Initial Idea

The PT formulated his/her initial idea *P*_0_(*n*) of the serious game, based upon his/her GTPACK. Textbox 2 shows what he wrote in the diary.

From the excerpts mentioned in Textbox 2, it can be inferred that the PT conceptualized his initial idea *P*_0_(*n*) of the serious game. He also defined the *N*=6 dimensions, where *n*=1 corresponds to the space (sprites: a source of the light ray, one mirror and one target)*, n*=2 denotes the dynamics (hidden objective, player autonomy, limited actions, and feedback), *n*=3 refers to mechanics (move, rotate, aiming and shooting, and allocating), *n*=4 corresponds to the sprite costumes (a torch, a line, a top view of a mirror, and a top view of a brick), *n*=5 refers to the aesthetics, and *n*=6 is for the programing dimension.

**Initial idea formulation-Diary excerpt**

*I can extend the image in the school textbook where there is a source that produces the light ray and mirrors at stable positions to reflect it so as through successive reflections it can reach a specific point. The students are asked to hypothesize the reflections and then draw a line to depict the path of the light ray as it reflects in this labyrinth of mirrors. Thus, if the student realizes this path once, then there is no interest to resolve it. So, I need to extend this activity towards a more interactive and interesting situation through my serious game. Keeping the analogy with the textbook activity, where it is presupposed that the student already has been taught the light mirror reflection, my educational aim is to involve the students in a serious game, in order to further elaborate the light reflection concept in more dynamic situations than the textbook, eg, the student can try the mirror reflection towards different lines of the ray and not in a specific labyrinth. Thus, my objective is to move the cognitive effort from the applying level and beyond according to the Bloom’s taxonomy. Moreover, I can use depictions from the textbook that the students are acquainted of, i.e., a torch as a source for the light ray, a line for the light ray, top view of a mirror and a top view of a brick as target.*

*I will elaborate on the well-known K-12 students’ misconceptions about the light reflection, concerning the equality of the angles of the light ray as it lands on and reflects from the mirror surface. I will assume that the students had already been taught the light mirror reflection; thus, they have conquered the first two levels of the Bloom’s taxonomy, so my serious game will not provide relevant information.*

*My objective is to move the cognitive effort concerning the aforementioned concept at the applying level of in the Bloom’s taxonomy.*

*As a tool I will use Scratch.*

*I will follow the GDM design framework and I will choose dynamics and mechanics from those presented in the work of [5], combined with additional important elements, namely:*

- *Space and Dynamics: The student should manage to apply the idea of the equal angles that are employed in the mirror reflection, but I do not want to show this; they must hypothesize the light line in a space arrangement that with include three sprites, ie, a source of the light ray, one mirror to reflect upon and one target where the reflected ray should hit, respectively. Thus, I choose the hidden objective dynamic. I also want the student to arrange in a way the game space, so I choose the player autonomy dynamic; yet, with limited actions so as not to be confused. Finally, I will provide feedback to his/her efforts, eg, a score counting the successive efforts toward the shooting the target with the reflected light ray.*
- *Mechanics: The torch will be at a stable position, but the student could rotate it. The mirror would be horizontally positioned in the bottom of the scene and could be moved in both directions on the x axis and the target will be at a stable position. Thus, I choose move, rotate, for the objects, aiming and shooting for the light ray, allocating for the hypothesized path of the light ray.*
- *Sprite costumes: I will use depictions from the textbook that the students are acquainted with, ie, a torch as a source for the light ray, a line for the light ray, top view of a mirror and a top view of a brick as target.*
- *Aesthetics: I think that the acquaintance of the students with the sprite costumes, the appealing scene, the explicit roles of the sprites and the challenge to target the brick will motivate the students to have fun and keep playing it toward higher scores. All these will contribute to the esthetics dimensions of my serious game, ie, its sensation (sense of pleasure), fantasy (make-believe), challenge (obstacle course), discovery (uncharted territory), and expression (self-discovery).*
- *Programming: I need to program all the needful with Scratch.*

### Dynamics in Adaptation Engine and Prototype Optimization

#### Overview

During the activity, the PT followed iteration paths through which a series of enhancement of the prototypes that were developed in Scratch resulted in the final output (the game). Here, indicative versions of the prototype are presented to reflect the dynamics and realization of the adopted optimization procedure within the 2D-ME framework. In particular, we followed the *P(n,k), n = 1,2,...,6, k = 1,2,...,5,* prototypes in the sequential order that they were developed, cumulatively constructing the serious game aspects. It should be noted that not all dimensions are simultaneously activated across the prototyping, because the PT placed the effort according to his temporal preferences and focus on specific dimensions.

#### Diary-Based Sampling

As a first means of dynamics acquisition, excerpts from the PT’s diary were used for each version of the prototype. Figure 5 facilitates the presentation at multiple levels that are used during the application of the 2D-ME framework, showcasing the activity system that the PT was involved in, its dynamics across the triangles’ vertices (Figures 3 and 5: {A,B,C},{a,b,c}), and the optimization procedure that took place. The corresponding activated dimensions (separated by semicolon) are described below; researcher SH’s external view based on the iAIS is also noted within brackets and included in Figure 5:

- Initial idea: 
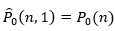
, Prototype: *P* (1;6,1)
  a→A: “From the first person view, I will start by setting the main scene and creating the first sprite; however, my GTPACK is not adequate to achieve this” (Question 1.2). “I need to enhance my GTK on how to program the reflection of the light ray on a moving mirror” (Question 3.2).
  A→C: “I need to incorporate multiple roles within myself to activate a reflective path for my design decisions” (Question 1.1 and Question 4.2).
  C→a: “From the first person view, I now feel more confident to start materializing my *P*_0_(*n*) idea” (Question 3.2).
  a→c: “From the first person view, I managed to write the Scratch code for the main scene and the first sprite and produce the first version of my object, ie, *P(1;6,1)*” (Question 1.4).
- Initial idea: 
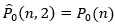
, Prototype:
  *P* (1; 4; 6, 2)
  a→b: “From the first person view, I will next put the mirror in the scene” (Question 1.2). “From the third person view, I realized that I need to revise my initial idea for the size and the way the mirror should look like, to avoid trivial game options” (Question 2.1).
  b→B: “The third person view helps me to identify design pitfalls and use one rule, i.e., to be on the shoes of the user, in order to better understand the game” (Question 4.1 and Question 4.2).
  B→a: “From the first person view, I keep as a rule to really play the prototype, in order to feel the pitfall that I conceived as such from the third person view” (Question 1.1 and Question 4.2). “I also follow the rule to distinguish between what remains the same and what should be changed towards optimization” (Question 1.4).
  a→c: “From the first person view, I understand that the third person view scaffolds my thoughts for the next step towards the implementation of my initial idea and I managed to write the Scratch code for the mirror, selecting the analogous size and view, producing the second version of my object, i.e., *P(1; 4; 6,2)*” (Question 1.4 and Question 3.4).
- Initial idea: 
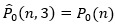
, Prototype: *P* (1; 3; 4; 6,3)
  a→b: “From the first person view, I will next put the brick and the light ray in the scene and adopt the sprite costumes of a line and top view of a brick, respectively” (Question 1.2). “From the third person view, I realized that I need to revise my initial idea for the fixed angle of the light source to avoid trivial game options” (Question 2.1).
  b→C: “I realized that I need to have a more organized sequence of reflections; hence, I have decided to adopt more frequent alternation between the first and third person view during my design process” (Question 4.2).
  C→a: “From the first person view, I now feel more excited to put more degrees of freedom to the torch” (Question 1.3).
  a→c: “From the 1st person view, I managed to write the Scratch code for the addition of the brick, the light ray and the rotation of the torch, producing the third version of my object, i.e., *P(1; 3; 4; 6,3)*” (Question 1.4).
- Initial idea: 
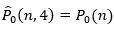
, Prototype: *P* (2; 3; 6,4)
  a→b: “From the first person view, I will next include the feedback to the student via a score display; however, I feel that something is missing...” (Question 1.2 and Question 2.1). “From the third person view, I had the idea of inserting randomness in the game. I find myself a bit strange when I have ideas that I do not know how to implement. It is a contradiction to my general attitude to proceed safely. However, when I follow them, I believe that new possibilities are revealed.” (Question 1.3, Question 2.1, and Question 2.2).
  b→A: “I feel that I need to expand my GTPK towards the motivational and engaging role of the surprises in the game through randomness and its relevant programming” (Question 3.2).
  A→C: “I can use my enhanced GTPACK and identify better the involvement of my different roles in myself for the technology- and aesthetics-related aspects of the prototyping” (Question 3.1, Question 4.1, and Question 4.2).
  C→a: “From the first person view, I broke down the move mechanic across my prototyping procedure, in order to realize which objects it should refer to, and materialize the decided randomness” (Question 1.2, Question 1.3, and Question 3.5).
  a→c: “From the first person view, I managed to write the Scratch code for the addition of the score and incorporate movement of the sprites, producing the fourth version of my object, i.e., *P(2; 3; 6,4)*” (Question 1.4).
- Initial idea: 
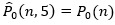
, Prototype: *P* (5; 6,5)
  a→b: “From the first person view, I feel that, most probably, I have reached the point of satisfaction with the structure of the game so far; but I am not 100% sure...” (Question 2.1). “From the third person view, I visualized a more interesting approach, focusing on the game aesthetics, that incorporated the sense of depth in the main scene” (Question 2.2 and Question 3.4).
  b→a: “From the 1st person view, I understood that this reflection provides me with many options to increase the sensation and fantasy of the game, using different 3D scene backgrounds” (Question 3.4).
  a→c: “From the first person view, I managed to write the Scratch code for the addition of a 3D background in the scene, producing the fifth version of my object, i.e., *P(5; 6,5)*” (Question 1.4). “I believe that, at this point, I have achieved my first higher goal to construct a good prototype of these functionalities and aesthetics of the game, converging to the initial idea conceptualized in the form of *P*_0_(*n*)” (Question 1.4 and Question 1.5).

It should be noted that in the excerpt of the optimization process presented earlier, the initial idea was kept constant across *k*, as the level of the game designing was of low complexity, trying to conceive and materialize the basic environment and functionalities of the game. As the game advanced, updates of the *P*_0_(*n*) took place, for example, through the insertion of >1 mirrors spreading randomly in the scene space and introduction of various game levels with different degrees of difficulty (eg, random distribution of nonreflecting obstacles and time limitation per level). Moreover, the GTPACK of the PT was enriched during the prototyping process, showcasing the internal dynamics that are developed across the design time at the GTPACK level, depicted as expanded overlapping spaces in Figure 5 (top). The estimation of the MSE (Figure 5, bottom) was based on the comparison (equation 1) between the current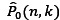 and the *P*(*n*,*k*) across the *n* dimensions. As the dimension of programing (*n*=6) is not included in the 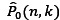, this was excluded from the MSE calculation, involving the dimensions of *n*=1,2,...,5 in equation 1. As at each *k*, not all dimensions are involved in *P*(*n*,*k*), the highest drop in the MSE is expected when many dimensions of *P*(*n*,*k*) are activated, as it can be seen at *k*=3 (*P*(1; 3; 4; 6,3)).

#### Prototype-Based Sampling

At a second sampling of the dynamics, with lower resolution, the materialization of the prototypes per optimization version were also analyzed. Figure 6 depicts the sequence of the produced prototypes for *k*=1,2,...,5, reflecting the specific dimensions that were examined at each *k*, visualized at the scene level and the corresponding Scratch code; the stimulus from the exercise textbook used as *P*_0_(*n*) is also presented. From Figure 6, at *k*=1, there is a very simple output at the scene level (one sprite); however, there is more extended structure at the programing level. This reflects the activation of the GTPACK as a means for extending the gaming programing background. At *k*=2, the work at the programing level is simplified and the scene is extended with the inclusion of the second sprite. The complexity of the programing is increased at *k*=3, because there is the addition of the third sprite and the light ray. This shows higher convergence to the initial idea, revealing that the PT follows an optimization process. In addition, at *k*=4, the experimentation with the light ray and the rotation of the sources (already seen at *k*=3), along with the addition of the user feedback (score) reveal an activation of the GTPACK (to enhance the game programing skills) and of the third-person view (also seen in Figure 5). In this way, the PT sees his prototype from the eyes of the user and tries to accommodate the issues of trivial solutions and lack of feedback, thus increasing the game options and esthetics. The interpersonal interaction about esthetics continues at *k*=5, in which the scene becomes 3D, providing more immersive impression to the user. Here, it seems that the dynamics between first- and third-person views are the ones that dominate (also seen in Figure 5), as the enhancement of the external (user’s) view of the game is the focus at this stage of prototyping.

## Discussion

### Principal Findings

The proposed 2D-ME conceptual framework surfaces the roles of the first- and third-person views that challenge the designer to realize aspects of his/her self in this iterative procedure. Being more reflective, the designer may realize that the self may be both a subject influenced by the society norms, for example, in the aforementioned case, the programing rules, but also a driving self that may influence the society through the output of their game design. These roles were reflected in the case study presented, which served as a showcase of the 2D-ME framework to reveal the dynamics within the iterative process of the prototype optimization, seen both from the designer’s space (diary) and the game design outcome (prototype). This may contribute to more knowledgeable game designers, as it provokes reflection in a fine-grained way of thinking at different levels. It should be noted that the case study presented did not entail the hierarchy of the AT framework and was kept mostly at the activity system level, focusing on the optimization procedure.

In the case study presented, the user is a PT, who had already acquired a basic knowledge of the GTPACK framework because he works in the education sector. Nevertheless, there is no requirement for someone to have any specific level of prior knowledge to use the 2D-ME framework; simply, someone who undertakes a prototype design process (in the specific case of game design) will most probably have a basic level of the TPACK in the field. Even if he/she is not an expert, during the prototyping process, he/she will interact with the knowledge base and would acquire extra knowledge in the area. This is schematically shown in both Figure 1C and Figure 5 (GTPACK), where the context of the GTPACK is not split in equal sections but there are different levels of knowledge within it (Figure 3, c), which are dynamically expanded during the interaction with the knowledge base (Figure 5; increased GTPACK for *k*=3→4). In addition, the knowledge of the iAIS is not provided as a constrain but as a guide to help the user formulate the internal reflection on the activities during the prototyping; someone could follow their own pathway of metacognitive process stimulation. However, we believe that the proposed iAIS targets all the reflective aspects that could be helpful to serve as a tool of memoir and reflexivity during prototyping.

Apart from focusing on the monitoring of the internal dynamics during the prototype optimization process, 2D-ME can also be used as a means of structure for the designer’s skills development in the reflection and creativity. Diary writing itself requires the designer to rethink events and processes, along with thoughts, contradictions, decisions, outcomes, which have all taken place at the various stages of the prototyping process, and “relive” it, as many times they want, even after its end. This strategy promotes their critical thinking and reasoning skills, that is, their higher-order thinking skills [39]. In addition, the stimulus from the iAIS (Textbox 1) sets an organized pathway of reflection steps that span across the triangle constructs involved in the 2D-ME framework. In this way, the activity dynamics are scaffolded by the iAIS stimulus and gradually become more imprinted in the game designer’s reflection strategy and higher-order thinking. When the narrative information of the diary is transformed into dynamics within the 2D-ME framework (Figure 5), a more externalized representation is achieved that could be visible, apart from the designer themselves, to others, as well. This can provide information for some characteristics of the designer’s personal way of thinking and creating, increasing their explainability, especially when they are not tangible at the prototype level and/or cannot be easily inferred from the final output (eg, the *P*(*n*,*M*)). In this way, the 2D-ME framework could provide more objectified basis for the analysis and comprehension of the expert designer’s process, from idea conception to the final output, which can be used for teaching the novice designers, help them to better understand their internal processes, and improve their designing skills and outcomes.

In the case study that was presented here, the prototyping process was optimized by taking into consideration the relation between 2 adjacent versions of the prototype, that is, for the creation of *P*(*n*,*k*), *P*(*n*,*k*–1), (*k*>1) was considered as input to the AT triangle (Figure 5). Nevertheless, additional views could also be adopted, and a higher (>1) order of memory could be used, for example, for the creation of *P*(*n*,*k*), *{P(n,k – 3), P(n,k – 2), P(n,k – 1)}, (k >3)* can be used as a sequence of inputs. Moreover, this sequence could also vary across the whole process, both in the number of the previous versions used and in their continuity, for example, *{P(n,k – 3), P(n,k –1)}, (k >3)*. This depends on the scale of the focus that is used at each *k*, providing the opportunity to the designer to shift from the micro- to the macroscale of the prototyping process [40,41]. This influence of previous prototype versions within the creation of the new one materializes the convolution of the dynamics adopted in the past with the dynamics shaped in the current version, in an effort to create a new, more enhanced prototype. This blending of dynamics in a macrostructural approach showcases the causalities that are created across the prototyping process that, in a way, contribute to the personal characteristics of the designer’s style. For example, sequential use of the prototyping versions (eg, as in Figures 5 and 6) could reveal steadier design style, in which each step is cumulatively constructed from the previous one. A more discontinued use of past prototyping versions could express more diffusion of the design ideas at various time instances of *k – l (l = 1,2,…*| *k – l >0)*, revealing a distributed way of materializing the design ideas, in which different parts of them gradually are synthesized and construct a more integrated prototype version at the *k*th time instance.

The adopted bilateral sampling of the dynamics in the 2D-ME framework, that is, via the analysis of the diary notes and the prototype versions, provides both the internal and external views of the prototyping dynamics and outcome, respectively. Additional means of dynamics sampling could also be foreseen by incorporating other sources of relevant information, such as biosignals. In fact, the biological-signal data from multiple biological-signal sources, for example, electroencephalography, electrocardiography, electromyography, electrooculography, and electrodermal activity, can be combined with artificial intelligence or machine learning or deep learning to provide additional queues of the developer’s state (eg, stress, relaxation, or hyper or hypoactivity) during the design of the game and the creation of a series of prototypes [42-44]. In this way, the projection of the creative thinking onto the physiological signals and testing of the same protocol to game users could reveal the transferability of the designer’s intentions to the gamers via the similarity of the related acquired signals. This could be used as a measure to optimize the game design and create a dynamic cocreation process [45-48], which could adapt to the specific characteristics of the designer and the target group of the serious game users [49-53].

### Limitations and Further Research

The proposed 2D-ME conceptual framework provides new insights into the internal dynamics of the game designer’s prototyping process. Clearly, the presentation here of 1 case study with 5 prototype versions cannot express the whole range and magnitude of such dynamics. However, it serves as a good example of the potentialities the 2D-ME framework could provide, both to the game designers and educators and researchers. In addition, the adoption of MSE (equation 1), as a cost function for the optimization process (Figure 4), could also be further explored, as alternative and a combination of metrics could be involved. The selection of such cost functions should consider the acceptable variation that relates with the context of a particular applied game design problem and should clearly express, as much as possible, the gains from its minimization under the game design problem’s particular circumstances [54]. Extensive experimental application of the 2D-ME framework (eg, in educational settings of game design) could further validate its efficiency to monitor the dynamics evolved within the game design prototyping process, not only under the controlled settings, as presented here, but also at more naturalistic environments, for example, at designers’ workplaces or laboratories.

The 2D-ME conceptual framework attempts to explain the self–first and self–third person views of prototyping dynamics in serious games design. Nevertheless, as it combines the TPACK and AT frameworks in a dynamic way, it shows many potentialities to be extended further to various design problems, additionally to the area of game design. In this regard, in our future plans, we foresee 2D-ME to be applied in the areas of arts, where the game design is replaced by art design. In many areas of art, such as painting, sculpture, literature, architecture, cinema, music, and theater, the design dynamics is the driving force for the creativity and the development of the artwork through prototyping. Clearly, the 2D-ME framework can easily embrace and explain such dynamics. Furthermore, sharing of the prototype version at specific *k* time instances to an external group (eg, experts, colleagues, mentors, and educators) could provide an additional level of external input to the internal world of the designer, extending the role of the community. In this vein, the current 2D-ME framework can be extended to the 2D-ME-ALL one, embracing external inputs and dynamics during the prototyping process—thus, enhancing the social and collaborative aspects in the use of the AT objects and extending to runaway ones with even broad societal ramifications [55]. Clearly, during the development of the prototype versions, externalization and projection from the “inner” to the “outer” world is possible and, under a cocreation perspective, wishful, to arrive at an optimized version. Moreover, this externalization does not have to be for the whole prototype but could be associated with specific parts (processes) of the prototyping, where external feedback is needed, upon the personal decision of the designer. In addition, it could happen during 1 or many iterations, providing a dynamic interplay between the inner and outer worlds. Approaching this as interconnected AT systems (constellations) [56] mainly shifts the locus of attention to the multitude of such internal activities, as externalized via their relationships, rather than the interplay between the self–first and self–third person views within the game designer’s mind. In this sense, the selection of the scale is important, and the inclusion of interconnected AT systems could explain a concept of collective contribution (ie, transition from the person to the group and, further, to society). Apparently, this can happen in the case of complementary contributions by different experts participating in the game prototyping, for example, directors, graphic designers, composers, light experts, and character creators. It also can be expanded to an interdisciplinary context in which the experts come from distant backgrounds (eg, health and technology). However, even in such cases, the 2D-ME framework could be adopted by each expert to describe the steps that they undertook to arrive at their final contribution to the game design. Finally, there are some creative processes that are performed alone by nature, for example, music composition and painting. In such cases, it is important to focus on the internal dynamics that take place in the artist, as, in this way, we can identify personal traits and idioms that finally characterize the personal artistic style. In this kind of cases, the constellation of AT systems could, probably, be adopted if we want to model the audience interaction with the artistic outcome, especially in cases where there is an interactive part in it, for example, music installations or interactive spaces (eg, Xenakis polytopes). These views and extensions of 2D-ME, along with the addition of biosignals’ input by incorporating artificial intelligence, machine learning, or deep learning tools, set the forthcoming research goals; work has already embarked toward such endeavor.

### Conclusions

A new conceptual framework, namely 2D-ME, that provides insights upon the dynamics that evolve during a serious game designer’s prototyping process has been presented here. Combining information from both the games space, TPACK, and AT, the 2D-ME framework provides explainable representation of, somehow, intangible processes; most of them not easily identifiable in the externalization of the prototyping process, that is, the prototype itself. Using dynamic constructs, the 2D-ME framework incorporates the alternations from first-person to third-person views and vice versa, stimulated by an organized way of internal self-interview (iAIS), providing dynamics monitoring that drive the prototype optimization process. A paradigm of the practical implementation of the 2D-ME framework in the case of a game design on light reflection exemplifies its potentialities and efficiency to capture the fine-grained dynamics involved during the production of the prototype version across the design timeline. Owing to its generic structure, the 2D-ME framework has high transferability to other areas of designing, for example, arts, and could be used both as a means of exploration of the expert designer’s creative process and training of the novice ones, providing explainable representations of the underlined higher-order thinking.

## Abbreviations

AT: activity theory
CK: content knowledge
GK: game knowledge
GPCK: game pedagogical content knowledge
GPK: game pedagogical knowledge
GTPACK: Games Technological-Pedagogical-Content Knowledge
iAIS: internal Activity Interview Script
MDA: Mechanics, Dynamics, and Aesthetics
MSE: mean square error
PCK: pedagogical content knowledge
PK: pedagogical knowledge
PT: preservice teacher
TCK: technological content knowledge
TK: technological knowledge
TPACK: Technological-Pedagogical-Content Knowledge
TPACK-G: Technological-Pedagogical-Content Knowledge–Game
TPK: technological pedagogical knowledge
